# Preparation and Evaluation of Animal Models of Cardiotoxicity in Antineoplastic Therapy

**DOI:** 10.1155/2022/3820591

**Published:** 2022-07-05

**Authors:** Chenchen Meng, Lu Fan, Xiaoming Wang, Yunjiao Wang, Yanyang Li, Shuchao Pang, Shichao Lv, Junping Zhang

**Affiliations:** ^1^First Teaching Hospital of Tianjin University of Traditional Chinese Medicine, Tianjin 300193, China; ^2^National Clinical Research Center for Chinese Medicine Acupuncture and Moxibustion, Tianjin 300193, China; ^3^Tianjin Medical University Cancer Institute and Hospital, Tianjin 300060, China; ^4^Tianjin Key Laboratory of Traditional Research of TCM Prescription and Syndrome, Tianjin 300193, China

## Abstract

The continuous development of antineoplastic therapy has significantly reduced the mortality of patients with malignant tumors, but its induced cardiotoxicity has become the primary cause of long-term death in patients with malignant tumors. However, the pathogenesis of cardiotoxicity of antineoplastic therapy is currently unknown, and practical means of prevention and treatment are lacking in clinical practice. Therefore, how to effectively prevent and treat cardiotoxicity while treating tumors is a major challenge. Animal models are important tools for studying cardiotoxicity in antitumor therapy and are of great importance in elucidating pathophysiological mechanisms and developing and evaluating modality drugs. In this paper, we summarize the existing animal models in antitumor therapeutic cardiotoxicity studies and evaluate the models by observing the macroscopic signs, echocardiography, and pathological morphology of the animals, aiming to provide a reference for subsequent experimental development and clinical application.

## 1. Introduction

**Figure 1:**
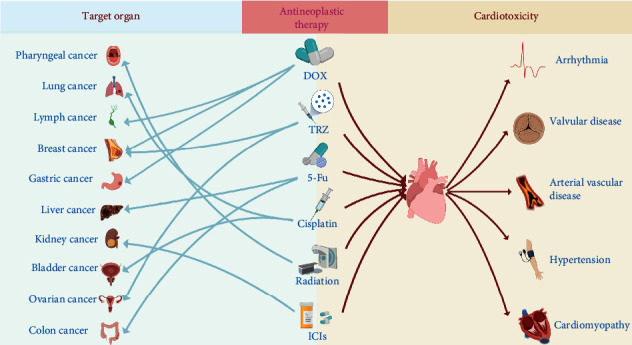
Diagram of the relationship between antineoplastic therapy and cardiotoxicity. The blue area on the left represents organ tumors treated with antineoplastic therapy, and the yellow area on the right represents cardiotoxicity resulting from antineoplastic therapy.

With the advancement of science and technology, modalities such as surgery, chemotherapy, radiotherapy, and targeted immunotherapy for the treatment of malignant tumors have been developed, which have greatly improved the survival rate of patients with malignant tumors. Statistics released by the American Cancer Society (ACS) show that the mortality rate of patients with malignant tumors has decreased by 31% since 1991 [1]. Although the survival rate of patients with malignancies has improved, cardiotoxicity associated with antineoplastic therapy has also become apparent. Studies have shown that the risk of cardiovascular death increases 1.6 to 3.6 times in patients after antineoplastic therapy, and the risk of cardiovascular risk factors such as hypertension, diabetes mellitus, and lipid metabolism disorders increases 1.7 to 18.5 times compared to the nononcology population [2]. Cardiotoxicity has become the leading cause of long-term death in patients with malignancies [3]. At this point, the emerging interdisciplinary discipline of oncology cardiology was born [4]. Oncologic cardiology focuses on cardiotoxicity caused by oncology treatment and oncology-combined cardiac diseases, aiming at the comprehensive, effective, and scientific management of patients with oncology-cardiology comorbidities and prevention of antitumor therapy cardiotoxicity [5]. Antineoplastic cardiotoxicity includes arrhythmias, arterial vascular disease, hypertension, and myocardial infarction (Figure 1), and the most common and serious ones are left ventricular dysfunction and heart failure [6]. Currently, for the prevention and treatment of antineoplastic therapy cardiotoxicity, some studies have proposed the use of cardioprotective agents to prevent antineoplastic therapy cardiotoxicities, such as dexrazoxane, coenzyme Q10, glutathione, antioxidants, leucovorin, N-acetylcysteine, and iron chelators, but the safety and efficacy of the above drugs are highly controversial. Dexrazoxane is the only cardioprotective agent approved by the Food and Drug Administration (FDA), and although many guidelines recommend its use for the prevention of anthracycline cardiotoxicity, some studies have found serious adverse effects, such as bone marrow suppression and neurotoxicity, in the use of dexrazoxane. Meanwhile, the 2008 U.S. oncology clinical practice guideline points out that dexrazoxane may reduce the antitumor efficacy of anthracyclines, thus limiting its clinical application and promotion [3, 7, 8]. In addition, there is a lack of evidence-based medical evidence for the role of other cardioprotective agents [9]. For the treatment of antitumor cardiotoxicity, the standard treatment for cardiac disease in nononcology patients is mainly followed, but this approach may not achieve the expected clinical efficacy because the cardiotoxic response in oncology patients with different disease processes and treatment regimens is clinically different from that in nononcology patients. For example, in an analysis of heart failure patients with ventricular assist devices, it was shown that patients with chemotherapy-related heart failure require more ventricular assist support devices than patients with other etiologies because chemotherapy causes secondary ventricular injury and triggers more severe heart failure [10, 11].

In conclusion, there is still a lack of practical measures to prevent and treat cardiotoxicity in antineoplastic therapy. The development of drugs that combine cardioprotective and anticancer effects has become a focus of research and a hot spot in recent years. Experimental research is inseparable from the replication of animal models, which can simulate the disease state, further explore the pathogenesis and pathological process, and provide a more scientific and reliable theoretical basis for the development of relevant drugs and clinical treatment. Therefore, it is important to establish animal models that are close to human cardiotoxicity in antitumor therapy. However, there are many uncertainties in the preparation of animal models, such as ambiguous modeling dose and preparation period. Therefore, this paper summarizes and evaluates the preparation methods of animal models for cardiotoxicity caused by antitumor therapy, aimed at establishing a clear and feasible animal model for cardiotoxicity of antitumor therapy and providing a basis for subsequent experiments and clinical applications.

## 2. Single-Factor-Induced Cardiotoxicity Animal Model

A single-factor-induced cardiotoxicity animal model refers to an animal model of cardiotoxicity replicated by an intervention factor, mostly established by injection of doxorubicin, trastuzumab, 5-fluorouracil (5-FU), cisplatin, immunosuppressive drugs, and radiation. And the model consistent with the clinical antitumor treatment cardiotoxicity was replicated and evaluated by observing the general state of the animal, cardiac ultrasound, and pathological morphology.

### 2.1. Animal Model of Doxorubicin Cardiotoxicity

Doxorubicin (DOX) is a highly effective, broad-spectrum anthracycline anticancer drug with very definite efficacy in the treatment of leukemia and many solid tumors, but time- and dose-dependent cardiotoxicity has limited its clinical application [12–17]. The pathogenesis of doxorubicin cardiotoxicity is still unclear, and current studies have only suggested its possible mechanisms, including the toxic effects of doxorubicin topoisomerase II, oxidative stress, and mitochondrial damage, and the lack of clarity on the mechanism also hinders the development of relevant preventive and curative drugs, so further studies are still needed by using animal models [18–20]. Regarding the construction of animal models of doxorubicin cardiotoxicity, according to domestic and foreign literature, most scholars chose mice, rats, rabbits, dogs, and zebrafish to construct animal models of doxorubicin cardiotoxicity by giving doxorubicin 5~45 mg/kg by intraperitoneal or tail vein injection. There are advantages and disadvantages to intraperitoneal or caudal intravenous administration. It has been found that although intraperitoneal administration is simple, it may cause peritoneal injury and increase the risk of noncardiac death, and peritoneal injury may also prevent the absorption of drugs in subsequent experiments, thus affecting the experimental results, while caudal intravenous administration can avoid the above problems, but it is difficult to operate and increases the risk of phlebitis and tail rot [21, 22]. In terms of the dose administered, some scholars believe that cumulative doses of doxorubicin at or below 10 mg/kg do not cause cardiotoxicity [23]; others suggest that a cumulative dose of 20 mg/kg is the lowest dose that can cause cardiotoxicity [24–26]; others have found that a cumulative dose of 24 mg/kg administered intraperitoneally can show both severe cardiotoxicities with a low mortality rate, which is more suitable for experimental studies [27].

Since doxorubicin cardiotoxicity is time- and dose-dependent, there are two types of animal models of doxorubicin cardiotoxicity: acute cardiotoxicity animal model and chronic cardiotoxicity animal model. Acute animal cardiotoxicity of doxorubicin usually occurs at the beginning of drug use, which is short-lived and reversible, with clinical manifestations appearing within 2 weeks after the end of treatment. Therefore, short-term, high-dose injections are generally used to establish doxorubicin acute cardiotoxicity animal models, which have the advantage of short modeling period and predictable time of cardiotoxicity but usually have high mortality rate and low model success rate, while chronic cardiotoxicity often occurs after long-term use of the drug, with clinical symptoms appearing within 1 year. Chronic cardiotoxicity often occurs after long-term use of the drug, and clinical symptoms appear within 1 year, so the low-dose, long-term injection of doxorubicin is generally chosen to replicate the chronic cardiotoxicity animal model of doxorubicin, which has the advantages of low mortality and long survival time of animals, and this mode of administration better simulates the clinical treatment regimen of intermittent dosing and the resulting chronic myocardial injury, but the experimental period is long and the time of the most obvious cardiotoxicity cannot be determined. The following sections describe the methods commonly used to prepare each of these two animal models.

#### 2.1.1. Animal Model of Acute Cardiotoxicity of Doxorubicin

A single intraperitoneal injection of DOX (10 mg/kg or 20 mg/kg or 25 mg/kg) or a single tail vein DOX (20 mg/kg) can be used to construct acute cardiotoxicity models in rats and mice [28–32], which showed symptoms such as reduced dietary intake, weight loss, diarrhea, reduced activity, decreased left ventricular ejection fraction, decreased left ventricular pressure change rate (± DP/DTmax), decreased -dP/dtmax, increased left ventricular end-diastolic pressure (LVEDP), myocardial fiber distortion and rupture, increased myocardial cell necrosis, increased type B (BNP), increased lactate dehydrogenase (LDH), and increased calponin T (cTnT); zebrafish embryos were placed in DOX at 30 *μ*M/100 *μ*M, and intraperitoneal injection of DOX (20 mg/kg) can replicate the zebrafish embryo and adult zebrafish models of acute cardiotoxicity of doxorubicin, which showed that doxorubicin can cause partial myocardial fiber arrangement disorder, cardiomyocyte sequestration, decreased left ventricular minor axis decoration rate (LVFS) and heart rate (HR), and increased serum BNP [33, 34].

#### 2.1.2. Animal Model of Chronic Cardiotoxicity of Doxorubicin

A rat model of chronic cardiotoxicity can be constructed by multiple intraperitoneal injections of DOX (cumulative doses of 10 mg/kg or 15 mg/kg or 24 mg/kg) and tail vein injections of DOX (cumulative doses of 6 mg/kg or 15 mg/kg), which showed myocardial cell edema, vacuolar degeneration, and myocardial fiber rupture in rats. The cardiomyocytes showed small focal or patellar necrosis. Left ventricular systolic pressure (LVSP), ±dp/dtmax, left ventricular diastolic dimension (LVIDD), FS, and ejection fraction (EF) decreased, while -DP/DTmax and LVEDP increased [28, 29, 35–37]. A rabbit model of chronic cardiotoxicity was established by multiple intravenous injections of DOX (cumulative doses of 16 mg/kg or 30 mg/kg) at the ear margins, which showed that rabbits were depressed, activity and food intake decreased, and rabbit hair fell off a lot [35, 38, 39]. Myocardial cells showed edema, degeneration, partial lysis, and necrosis. LVEDP and left ventricular end-diastolic dimension (LVDD) increased, while left ventricular systolic pressure (LVSP) and ±dp/dtmax decreased. The contents of cardiac troponin I (cTnI) and BNP increased. A canine model of cardiotoxicity was established by using cephalic intravenous DOX (cumulative dose 9.25-13.75 mg/kg or 240 mg/m^2^), which showed canine cardiomyocyte vacuolation, decreased FS and left ventricular ejection fraction (LVEF), increased LVIDD and left ventricular end-systolic diameter (LVESD), and increased serum BNP levels [40, 41]. In addition, a porcine model of cardiotoxicity was replicated by using multiple coronary injections of DOX (cumulative dose of 100 mg), whose results showed that the left ventricular work (LVSW) and left ventricular stroke work index (LVSWI) decreased in pigs [42].

### 2.2. Animal Models of Trastuzumab Cardiotoxicity

Trastuzumab (TRZ) is a humanized monoclonal antibody that recognizes human epidermal growth factor receptor-2 (HER-2). It produces antitumor activity by specifically binding to the HER-2 oncogene expression product P185 protein on tumor cell membranes [43]. In 1998, the FDA-approved trastuzumab for the treatment of invasive breast cancer with HER2 gene overexpression results in a 50% and 33% reduction in recurrence and mortality rates, respectively [44]. However, subsequent clinical trials and dosing revealed a high incidence of cardiotoxicity, which was mainly manifested by reduced left ventricular systolic function, asymptomatic heart failure, and heart failure with symptoms and signs [45, 46]. The molecular mechanism of trastuzumab-induced cardiotoxicity is still unclear, and some studies have found correlations between the renin-angiotensin system, NADPH oxidase, mitogen-activated protein kinase (MAPK) signaling pathways, MAPK/ERK1/2 phosphatidylinositol 3 kinase/Akt, and FAK-dependent cell survival signaling and HER2 receptor signaling pathways, but the connection is not clear; therefore, it needs to be further explored using animal models [25, 47, 48]. According to the existing literature, most scholars use rats and rabbits for the establishment of animal models of trastuzumab cardiotoxicity, because compared with mice, rats and rabbits have more mature techniques for cardiac function studies, and cardiac function is less affected by anesthesia and other intervention factors, which makes it easier to obtain more accurate experimental data; in terms of the mode of administration, since trastuzumab is mostly administered by slow intravenous drip, it is easier to simulate the effect of prolonged entry of trastuzumab into the circulatory system by peritoneal and subcutaneous administration than by intravenous administration. Therefore, some scholars have successfully replicated trastuzumab cardiotoxicity models by using rats and rabbits with cumulative doses of 15-60 mg/kg administered intraperitoneally or subcutaneously and evaluated the models by cardiac ultrasound and pathological morphology.

A rat cardiotoxicity model was constructed by multiple intraperitoneal injections of trastuzumab (cumulative doses of 15.75 mg/kg or 48 mg/kg or 60 mg/kg), which showed myocardial fibrosis, decreased LVEF and FS, increased LVDD and end-systolic volume (ESV), and increased serum LDH and cTnI levels in rats [49–51]. The subcutaneous injection of trastuzumab (cumulative dose of 26 mg/kg) replicated the cardiotoxicity model in rabbits, which showed lymphocyte and macrophage infiltration around myocardial cells and decreased LVEF, suggesting a decrease in left ventricular function in rabbits [52].

### 2.3. Animal Model of 5-Fluorouracil Cardiotoxicity

5-Fluorouracil (5-Fu) is an antimetabolic anticancer agent that is widely used in chemotherapy, especially in gastrointestinal tumors. However, in recent years, more and more clinical cases have shown the serious cardiotoxic side effects of 5-Fu during its application, and the incidence of cardiotoxicity is second only to anthracycline antibiotics [53–55]. Currently, the main mechanisms of 5-Fu cardiotoxicity include coronary artery spasm, endothelial injury-induced thrombosis, and oxidative stress, but these proposed mechanisms are based on only a few small experimental studies, and there are no uniform criteria for the diagnosis and prevention of 5-Fu cardiotoxicity so we require in-depth research with activity models [56–62]. According to the existing literature, most scholars have used single or multiple intravenous injections to replicate 5-Fu cardiotoxicity animal models, which are more in line with the clinical application but increase the risk of phlebitis; the experimental animals are mostly rabbits and rats, which facilitate better observation of cardiac changes.

Some researchers used multiple intravenous injections of 5-FU (cumulative dose 40 mg/kg), multiple intraperitoneal injections of 5-FU (cumulative dose 300 mg/kg), or single intraperitoneal injections of 5-FU (cumulative dose 150 mg/kg) to replicate cardiotoxic rat models, and the results showed that the rats showed symptoms such as depression, severe diarrhea, and loss of appetite. Extensive separation and distortion of myocardial fibers, accompanied by inflammatory cell infiltration around the cells, creatine kinase (CK), C-reactive protein (CRP), the levels of CRP, tumor necrosis factor-*α* (TNF-*α*), and interleukin-1*β* (IL-1*β*) increased, suggesting that 5-FU can cause myocardial injury in rats [63–65]. Other scholars used a single intravenous injection of 5-FU (50 mg/kg) and multiple injections of 5-FU (cumulative dose of 60 mg/kg) to establish a rabbit model of cardiotoxicity, and the results showed that the rabbit left ventricular wall had a large area of hemorrhagic infarction, myocardial cells showed multifocal necrosis, and the left ventricular wall increased, suggesting left ventricular dysfunction [66].

### 2.4. Animal Models of Cisplatin Cardiotoxicity

As a broad-spectrum cytotoxic drug, cisplatin is inexpensive and highly effective and is commonly used to treat advanced bladder cancer and other malignancies, but its induced cardiotoxicity has received much attention in recent years [67]. Existing studies have confirmed that the mechanisms of cisplatin cardiotoxicity include cytotoxic effects of cisplatin, oxidative stress, and inflammation, but the specific mechanisms of action are not well defined, and there is no definite drug proven to be a protective agent against cisplatin cardiotoxicity, so further studies using animal models are needed. The amount of literature on the establishment of animal models of cisplatin cardiotoxicity is very small, and more studies are needed for refinement.

Some researchers used multiple intraperitoneal injections of cisplatin (cumulative dose of 12 mg/kg or 120 mg/kg) and a single intraperitoneal injection of cisplatin (cumulative dose of 7 mg/kg) to replicate the cisplatin cardiotoxicity mouse model, which showed myocardial fiber degeneration and rupture, myocardial cell edema and vacuole-like degeneration, increased myocardial apoptosis, and increased creatine kinase isoenzyme (CK-MB), LDH, and cTnI content, suggesting that cisplatin can cause myocardial injury in mice [23, 24, 27, 30, 38, 39, 65, 68–126]. A single intraperitoneal injection of cisplatin (cumulative dose of 20 mg/kg) was selected to replicate a rat model of cisplatin cardiotoxicity, which showed an increase in cTnI and LDH content, suggesting myocardial damage in rats [70].

### 2.5. Animal Model of Radiation Cardiotoxicity

Radiotherapy significantly reduces mortality in thoracic malignancies and plays a pivotal role in the treatment of tumors. However, the radiation heart damage (RIHD) induced by radiotherapy increases the mortality of tumor patients to a certain extent [71, 72]. Some studies have shown that the risk of heart injury increases by 7.4% when the radiation dose increases by 1 Gy [73]. As for the pathogenesis of RIHD, it mainly includes vascular injury, endothelial dysfunction, oxidative stress, etc., but most of the studies on the above mechanisms only involve one or two pathways, which are not comprehensive and in-depth and need further research [74, 75]. As for the prevention and treatment of RIHD, although the cardiac radiation dose continues to decrease with the continuous development of radiotherapy technology, which reduces the risk of RIHD to a certain extent, this only plays a preventive role, and no effective drugs have been developed for the treatment of RIHD. How to clarify its molecular mechanism and effectively treat RIHD needs to be continuously explored by using relevant animal models [76]. According to existing literature reports, RIHD model animals are mainly rats, mice, rabbits, dogs, and monkeys [77–86]. Some scholars believe that single local irradiation with a dose of 15~25Gy should be selected for rats to establish an irreversible RIHD animal model. For larger animals (rabbits, monkeys, etc.) than rats, a single dose of more than 20 Gy should be selected [87]. In terms of irradiation methods, single or multiple local irradiation, whole-body irradiation, and dose division irradiation can be used [86, 88]. Most scholars choose single or multiple local irradiations, because compared with the latter two irradiation methods, it has a precise site of action and high clinical applicability and is more scientific, reasonable, and standardized.

The RIHD rat model was established by single local cardiac irradiation at a total dose of 15, 18, and 20 Gy or multiple local cardiac irradiations (cumulative dose of 45 Gy) [77–79, 89], which showed anorexia, hair loss in the cardiac projection area, physical wasting, slow activity, cardiac myocyte congestion and edema with inflammatory cell infiltration, myocardial tissue fibrosis, increased left ventricular end-diastolic posterior wall (LVPDW), and increased cTnI levels; a rabbit model of RIHD could be replicated using a single local cardiac irradiation at a total dose of 10-54 Gy, which showed collagen fiber proliferation, focal necrosis of cardiomyocytes, a large number of inflammatory cell infiltrates, and elevated cTnI content, suggesting radiation therapy-induced myocardial injury in rabbits [80].

### 2.6. Animal Models of Immunosuppressant-Induced Cardiotoxicity

Immune checkpoint inhibitors (ICIs) are the most promising antitumor therapies and have made significant advances in the treatment of advanced tumors such as progressive melanoma, renal cell carcinoma, and non-small-cell lung cancer [90]. However, several cases of severe cardiotoxicity caused by ICIs have recently been reported in international authoritative journals; meanwhile, some studies have shown that ICIs may cause fulminant progression of myocarditis and thus lead to patient death, thus receiving widespread attention [91]. However, the mechanism of action of immune checkpoint inhibitor-induced cardiotoxicity has not been fully elucidated, and there is a lack of uniform indicators for monitoring related cardiotoxicity, and preventive and related therapeutic measures have yet to be developed, all of which require animal models for relevant studies. Currently, the main immune checkpoint inhibitors used in clinical antitumor therapy include programmed cell death protein 1 (PD-1) inhibitors, programmed cell death ligand protein-1 (PD-L1) inhibitors, and programmed cell death protein 1/cytotoxic T lymphocyte-associated antigen 4 (PD-1/CTLA-4) inhibitors on which existing studies focus. The construction methods of ICI cardiotoxicity animal models include two main categories: gene knockout and drug injection. Gene knockout is mainly to knock out PD-1, PD-L1, and CTLA-4 genes; drug injection mainly includes intraperitoneal injection of the CTLA-4 inhibitor ipilimumab, intraperitoneal injection of the PD-1/PD-L1 inhibitor BMS-1, and tail vein injection of PD-L1 antibody and anti-PD-1 antibodies. Knockout-constructed cardiotoxicity animal models are more able to achieve the purpose of accurate research, while excluding the influence of other experimental factors, such as the absorption and metabolic process of drugs, and the research results are accurate and reliable, but they are operationally difficult. They mostly choose mice as experimental animals, because the genome sequencing program of mice has been completed and the genome modification technology is mature. The animal model of cardiotoxicity constructed by drug injection can better simulate the clinical use of immune checkpoint inhibitors and is simple to operate.

The ICI cardiotoxicity mouse model could be established by knocking out the PD-1/CTLA-4 gene, and the results showed that the mouse cardiomyocytes were deformed, myofilaments were disordered and broken, mitochondria were irregularly shaped, there were a large number of lymphocytes and multinucleated cells infiltrated between cells, the content of cardiac inflammatory markers interleukin-2 (IL-2) and TNF-*α* was increased, LVDS and LVDD were elevated, and FS was decreased, suggesting left ventricular dysfunction and myocardial injury in mice. However, one study found that mating knockout PD-1 mice with knockout CTLA-4 mice produced Ctla4 ^+/-^ Pdcd1 ^−/−^ offspring mice that were more suitable for ICI myocarditis studies, and such mice showed more pronounced and clinically similar changes in cardiac injury relative to simultaneous knockout PD-1/CTLA-4 mice [92–95]. Replication of the ICI cardiotoxicity mouse model using intraperitoneal injection of BMS-1 (cumulative doses of 30 mg/kg or 60 mg/kg) showed interstitial fibrosis of cardiomyocytes, decreased body weight, increased heart-to-body mass ratio, and increased serum levels of cardiac markers such as BNP, CK-MB, and LDH and increased proapoptotic proteins such as caspase-3 and caspase-9 levels, indicating cardiac injury in mice [122]. The ICI cardiotoxicity mouse model was established by multiple intraperitoneal injections of ipilimumab (cumulative dose 105 mg/kg), which showed decreased FS and radial strain (RS) and increased cardiac inflammatory markers IL-2 and TNF-*α*, indicating decreased cardiac function and damaged cardiomyocytes in mice [97]. The reproducible ICI cardiotoxicity mouse model by multiple tail vein injections of anti-PD-L1 antibody (cumulative dose 60 *μ*g/g) and anti-PD-1 antibody (cumulative dose 75 *μ*g/g) showed that mice were huddled and immobile, with significantly reduced response to external stimuli, cardiomyocyte hypertrophy, intercellular lymphocyte and neutrophil infiltration, and reduced EF, FS, and LVEDV, suggesting that mice' left ventricular function is reduced [98, 99]. In addition, the cardiotoxic crab monkey model can be replicated using multiple intravenous administrations of nabumab (cumulative dose 80 mg/kg) and epirubicin (cumulative dose 60 mg/kg), which showed a large infiltration of monocytes around the cardiomyocytes of crab monkeys, and cardiac inflammatory markers interleukin-6 (interleukin 6, IL-6), gamma interferon-*γ* (IFN-*γ*), and TNF-*α* were increased, suggesting myocardial injury in crab-eating monkeys [100].

## 3. Animal Models of the Compound-Induced Cardiotoxicity

Animal models of the compound-induced cardiotoxicity refer to an animal model of cardiotoxicity induced by a combination of antitumor modalities. Since single antitumor therapy does not achieve the desired effect and is prone to drug resistance, in contrast, combination therapy can enhance the therapeutic effect while overcoming drug resistance [90, 101]. Therefore, the combination therapy model is more in line with the clinical situation than monotherapy, but it can induce more severe cardiotoxicity with a superimposed effect on cardiotoxicity [101, 102]. The main combination treatment modalities that commonly cause severe cardiotoxicity in clinical practice are doxorubicin combined with trastuzumab and radiation combined with trastuzumab, and most of the existing studies have been conducted around these two aspects.

### 3.1. Doxorubicin Combined with Trastuzumab Cardiotoxicity Animal Model

Doxorubicin (DOX) in combination with trastuzumab (TRZ) is commonly used as a standard chemotherapy regimen for the clinical treatment of human epidermal growth factor receptor 2- (HER-2-) positive breast cancer, and its efficacy is remarkable and well tolerated by patients [103–106]. However, trastuzumab and doxorubicin are both highly cardiotoxic, and some studies have shown a superimposed effect of combination-induced cardiotoxicity [107, 108]. Regarding the establishment of DOX combined with TRZ cardiotoxicity animal models, most scholars adopted single intraperitoneal injections of DOX (20 mg/kg) and TRZ (10 mg/kg) or multiple intraperitoneal injections of DOX (cumulative dose 15-24 mg/kg) and TRZ (cumulative dose 10-30 mg/kg) and observed the general status of animals, cardiac ultrasound, pathological morphology, etc. to evaluate the animal model.

A mouse model of cardiotoxicity can be established by simultaneous intraperitoneal injection of DOX 20 mg/kg, TRZ 10 mg/kg or DOX 6 mg/kg, and TRZ 10 mg/kg or DOX (cumulative dose of 24 mg/kg) for 1 week followed by TRZ (cumulative dose of 10 mg/kg), whose results showed that the myocardial cells of mice were altered by myofibrillar degeneration and vacuolization and infiltrated by a large number of surrounding inflammatory cells, LVESV and LVEDD were increased, LVEF and FS were decreased, and serum CK-MB and cTnI levels were increased, suggesting myocardial injury in mice [24, 27, 30, 109–111]. The cardiotoxic rat model was induced by intraperitoneal injection of DOX (cumulative dose 15 mg/kg or 20 mg/kg), TRZ (cumulative dose 20 mg/kg), and DOX (cumulative dose 15 mg/kg) at 8 days, TRZ (cumulative dose 20 mg/kg) and DOX (cumulative dose 20 mg/kg) after 11days, and then TRZ (cumulative dose 30/kg) after 2 weeks, whose results showed that there were a large number of macrophages around the rat cardiac cells, left ventricular end-systolic volume (LVESV) and left ventricular end-diastolic volume (LVEDV) increased, FS and longitudinal strain (LS) decreased, and serum cTnI and N-terminal probrain natriuretic peptide (NT-proBNP) levels increased [112, 117, 120].

### 3.2. Radiation Combined with Trastuzumab Cardiotoxicity Animal Model

Like DOX combined with TRZ, radiation combined with trastuzumab is also commonly used in clinical practice to treat HER-2-positive breast cancer, with the difference that it is not clear whether the simultaneous application of radiation therapy with TRZ superimposes its cardiotoxicity [21]. Therefore, it is necessary to investigate the degree of cardiotoxicity induced by radiation combined with TRZ and its mechanism of action using animal models. According to the existing literature, some scholars constructed a RIHD rat model by single local irradiation of the heart at 15 Gy combined with a single intraperitoneal injection of TRZ (6 mg/kg), which showed mitochondrial edema and endothelial vacuole-like changes in rat cardiomyocytes, suggesting myocardial injury in rats [77]. Some scholars also used single local irradiation of the heart at 15 Gy or 20 Gy combined with multiple intraperitoneal injections of TRZ (cumulative dose 10 mg/kg) to replicate the RIHD mouse model, which showed a decrease in body weight, disturbed arrangement of cardiomyocytes with vacuolar and fat-like changes, and increased left ventricular posterior wall thickness (LVPWT) and interventricular septal thickness (IVST), suggesting a decrease in LV function in mice [83].

## 4. Application of Animal Models of Cardiotoxicity in Antitumor Therapy for Mechanism Exploration and Drug Development

### 4.1. Application of Animal Models of Cardiotoxicity in Antitumor Therapy for Mechanism Exploration

In recent years, based on animal models of cardiotoxicity in antitumor therapy, breakthroughs have been made in the study of related cardiotoxicity mechanisms. For example, Fang et al. found that iron death is one of the key mechanisms of adriamycin cardiotoxicity by using the mouse model of adriamycin cardiotoxicity, which provides a novel strategy for the prevention and treatment of adriamycin cardiomyopathy [113]; Zhang et al. found that DNA topoisomerase IIB (TOP2B) is an important target for the occurrence of adriamycin cardiotoxicity by using the mouse model of adriamycin cardiotoxicity [114]; Chen et al. used the PD-1/PD-L1 inhibitor BMS-1 to construct a mouse model of immune checkpoint inhibitor-associated cardiotoxicity and found that abnormal gut flora function may be one of the mechanisms of PD-1/PD-L1 inhibitor-associated cardiotoxicity and suggested that targeting gut flora to inhibit M1 polarization of colonic macrophages is a potential therapeutic strategy for PD-1/PD-L1 inhibitor-associated cardiotoxicity. The discovery of these mechanisms and targets of action has laid a solid foundation for the development of related drugs [96].

### 4.2. Application of Animal Models of Cardiotoxicity in Antitumor Therapy for Drug Development

Animal models of antitumor therapeutic cardiotoxicity are also of high application in the development of relevant drugs for prevention and treatment. So far, most of the studies related to the prevention and treatment drugs recommended in various guidelines for the management of cardiotoxicity in antitumor therapy have used animal models, such as statins, ACEI, and ARB. Using the adriamycin cardiotoxicity mouse model, Li et al. found that statins significantly improved cardiomyocyte injury in mice through antiapoptotic effects and proposed that statins could be used as a DOX-induced cardiotoxic protective agent [99]; Kabel et al. and Adeneye et al. using a rat model of trastuzumab cardiotoxicity, respectively, found that the combination of rosuvastatin with ubiquinone and antihypertensive drugs (valsartan, amlodipine, and lisinopril) has great potential in the prevention and treatment of trastuzumab-induced cardiotoxicity [115, 116].

In addition, animal models have also played an important role in the exploration of novel antitumor therapeutic agents for the prevention and treatment of cardiotoxicity. For example, Li et al. used a mouse model of doxorubicin cardiotoxicity to find that thrombopoietin (TPO) significantly attenuated cardiotoxicity in mice through antioxidant and anti-inflammatory effects, providing a new option for the treatment of doxorubicin cardiotoxicity [99]; Milano et al. used a rat model of cardiotoxicity induced by the combination of doxorubicin and trastuzumab to find that miR-146a-5p-mediated human CPC exosomes attenuated Dox-coinduced oxidative stress injury in the heart [117]; Chen et al. used the anti-PD-1 antibody and anti-PD-L1 antibody cardiotoxic mouse model and found that levothyroxine had a significant alleviating effect on PD-1/PD-L1-induced cardiotoxicity.

## 5. Summary and Conclusion

Animal models, as the experimental basis of experimental and clinical hypotheses, have become an extremely important experimental method and means in modern biomedical research, which not only overcomes the time and space limitations of clinical empirical research but also avoids many moral and methodological limitations of many experiments, and the establishment of scientific and effective experimental animal models is the basis for the study of pathogenesis and preventive and curative drugs. Currently, most scholars have chosen rats, mice, and rabbits as the models for the preparation of animal models of antitumor therapeutic cardiotoxicity, and the modeling methods are drug injection and targeted gene knockout (Table 1), which have great application value in elucidating the mechanism of antitumor therapeutic cardiotoxicity and the development of related prevention and treatment drugs, but there are also many problems. (1) The intervention dose is vague and very controversial. For example, regarding the ideal intervention dose of doxorubicin in the preparation of doxorubicin cardiotoxicity animal models, some scholars proposed that 15 mg/kg is the ideal intervention dose as it is close to the cumulative dose of clinical doxorubicin use and can clearly show cardiotoxicity, but some scholars found that the cumulative dose of 20 mg/kg is the lowest dose that can cause cardiotoxicity [24–26, 37]. (2) The modeling period is not clear, and some antitumor therapy cardiotoxicity has a delayed character, some even manifest several years after treatment, such as radiation cardiotoxicity, and most scholars often conduct different studies for multiple periods to determine the optimal modeling period, which consumes a lot of energy and material power and hinders the progress of experiments to some extent. (3) Animal models cannot fully simulate the human disease process. For example, current clinical experiments have found that trastuzumab-induced cardiotoxicity is reversible, while animal models of trastuzumab cardiotoxicity can only show the manifestation of trastuzumab-induced cardiomyocyte damage after the reversible process [96, 118, 119]. Therefore, how to optimize the existing animal models of antitumor therapy cardiotoxicity, as well as to further study and improve the production methods of animal models of antitumor therapy cardiotoxicity, and how to establish animal models with simple operation, high survival rate, good stability, and better simulation of the characteristics of human antitumor therapy cardiotoxicity are still the key and difficult problems for future research in this field.

## Figures and Tables

**Table 1:** Summary of preparation methods of animal models of antitumor cardiotoxicity.

Interference factors	Animal	Preparation methods	General situation	Cardiac structure and function	Myocardial injury	Pathological changes of the myocardium	Ref.
Chemotherapy							
DOX	Mice	Single intraperitoneal injection of 25 mg/kg	—	LVEF, FS↓	Myocardial disorder and interstitial edema	LDH↑, CK-MB↑	[33]
DOX	Mice	Multiple intraperitoneal injections of 4 mg/kg/w with a cumulative dose of 24 mg/kg	Weight loss	LVESV, LVEDV↑, LVEF↓	Myocardial fibrosis interstitial and collagen deposition	cTnI↑	[29]
DOX	Rat	A single intraperitoneal injection of 10 mg/kg, 20 mg/kg, or 20 mg/kg in the tail vein	Decreased diet and activity, weight loss, diarrhea	LVEF, ±dp/dtmax↓, -dP/dtmax, LVEDP↑	Myocardial fibers are twisted and broken, and myocardial cells are necrotic	BNP, LDH, cTnT↑	[30–32, 34]
DOX	Rat	Multiple intraperitoneal injections of 4 mg/kg/w with a cumulative dose of 24 mg/kg and 1 mg/kg/w with a cumulative dose of 15 mg/kg or 1 mg/kg/w with a cumulative dose of 6 mg/kg were given through the tail vein	—	LVSP, ±dp/dtmax, LVIDD, FS, EF↓, -dP/dtmax, LVEDP↑	Myocardial cell edema, vacuolar degeneration, myocardial fiber fracture, myocardial cell necrosis	—	[38, 39, 64]
DOX	Rabbit	Multiple intraperitoneal injections of 1 mg/kg/w and 2 mg/kg/w with a cumulative dose of 16 mg/kg or 3 mg/kg/w and a cumulative dose of 30 mg/kg	Loss of spirit, decreased diet and activity, a large number of rabbit hair loss	LVEDP, LVDD↑, LVSP, ±dp/dtmax↓	Myocardial cells are edematous and degenerative, a part of which is dissolved to necrosis	cTnI, BNP↑	[40, 41, 127]
DOX	Dog	Multiple cephalic intravenous injections of 1.5 mg/kg/3 w with a cumulative dose of 9.25-13.75 mg/kg or multiple cephalic intravenous injections of 30 mg/m^2^/3 w with a cumulative dose of 240 mg/m^2^	—	FS, LVEF↓, LVIDD, LVESD↑	Myocardial cells are vacuolar	BNP↑	[42, 45]
DOX	Zebrafish	Single intraperitoneal injection of 25 mg/kg	—	FS, HR↓	Myocardial fiber arrangement disorder, myocardial cell pyknosis	BNP↑	[35]
DOX	Zebrafish embryos	Put the embryos in DOX at the concentration of 30 *μ*M/100 *μ*M	—	FS, HR↓	Severe pericardial edema and distorted	—	[36]
DOX	Pig	Coronary artery injection 25 mg/w with a cumulative dose of 100 mg	—	LVSW, LVSWI↓	—	—	[46]
5-FU	Rat	Multiple intravenous injections of 8 mg/kg/d with a cumulative dose of 40 mg/kg or multiple intraperitoneal injections of 50 mg/kg/w with a cumulative dose 300 mg/kg or single intraperitoneal injections of 150 mg/kg	Depression, severe diarrhea, loss of appetite	—	Myocardial fibers are extensively isolated and distorted, and the myocardial cells are surrounded with inflammatory cells	CK, CRP, TNF-*α*, IL-1*β*↑	[63, 64, 67]
5-FU	Rabbit	A single intravenous injection of 50 mg/kg or 15 mg/kg/w with a cumulative dose of 60 mg/kg	—	LVWT↑	The cardiomyocytes show multifocal necrosis and are infiltrated with lymphocytes and neutrophils	—	[68]
Cisplatin	Mice	Multiple intraperitoneal injections of 4 mg/kg/2 d with a cumulative dose 12 mg/kg or 20 mg/kg/2 d and a cumulative dose 120 mg/kg	—	—	Myocardial fiber degeneration and rupture, myocardial cell edema and vacuolar degeneration, myocardial cell apoptosis increased	CK-MB, LDH, cTnI↑	[71, 72]
Cisplatin	Rat	A single intraperitoneal injections of 20 mg/kg	—	—	—	cTnI, LDH↑	[70]
Targeted therapy							
TRZ	Rat	Multiple intraperitoneal injections of 2.25 mg/kg/d with a cumulative dose of 15.75 mg/kg or 6 mg/kg/d and a cumulative dose of 48 mg/kg or 20 mg/kg/w, 60 mg/kg	—	LVEF, FS↓, LVDD, ESV↑	Myocardial fibrosis	LDH, cTnI↑	[51–53]
TRZ	Rabbit	The first dose of multiple subcutaneous injections was 8 mg/kg, and the cumulative dose was 26 mg/kg	—	LVEF↓	Numerous lymphocytes and macrophages are infiltrated around the myocytes	—	[54]
Radiation therapy							
Ray	Rat	Single local cardiac irradiation with a total dose of 15, 18, and 20 Gy	Anorexia, depilation in the projection area of the heart surface, emaciation, slow movement	LVPDW↑	Myocardial tissue fibrosis, myocardial cell hyperemia edema with inflammatory cell infiltration	cTnI↑	[79–81]
Ray	Rat	Multiple local cardiac irradiation was 9 Gy/d, and the cumulative dose was 45 Gy	—	—	Deposits of type I and III collagen are increased in cardiomyocytes	—	[22]
Ray	Rabbit	Single local cardiac irradiation with a total dose of 10~54 Gy	—	—	Myocyte collagen fiber hyperplasia, focal necrosis of myocyte, intercellular infiltration of a large number of inflammatory cells	cTnI↑	[82]
Immune checkpoint inhibitor therapy							
Gene knockout	Mice	Specific excision of the PD-1/CTLA-4 gene	—	FS↓, LVDS, LVDD↑	Myocardial fiber degeneration and rupture, numerous lymphocytes and multinucleated cells infiltrating in intercellular space	IL-2, TNF-*α*↑	[97, 98, 118]
BMS-1	Mice	Multiple intraperitoneal injections of BMS-1 with a cumulative dose of 30 mg/kg and 60 mg/kg	Weight loss	—	—	BNP, CK-MB, LDH↑	[96]
Ipilimumab	Mice	Multiple intraperitoneal injections of ipilimumab 15 mg/kg/3d with a cumulative dose of 105 mg/kg	—	FS, RS↓	—	IL-2, TNF-*α*↑	[101]
Anti-PD-L1 antibody	Mice	Anti-PD-L1 antibody was injected 10 *μ*g/g/w or 10 *μ*g/g/5 d by tail vein multiple times, and the cumulative dose was 60 *μ*g/g	Crouching silently, significantly weakened response to external stimuli	EF, FS, LVEDV↓	Cardiomyocyte hypertrophy, numerous lymphocytes and neutrophil cells infiltrating in intercellular space	—	[102]
Nivolumab and ipilimumab	Cynomolgus monkey	Multiple simultaneous intravenous injections of 20 mg/kg/w nivolumab with a cumulative dose of 80 mg/kg and 15 mg/kg/w ipilimumab with a cumulative dose of 60 mg/kg	—	—	There are numerous monocytes infiltrating around the cardiomyocytes	IL-6, IFN-*γ*, TNF-*α*↑	[89]
Chemotherapy combined with targeted therapy							
Dox in combination with TRZ	Mice	(1) Intraperitoneal injection of DOX 20 mg/kg, TRZ 10 mg/kg or DOX 6 mg/kg, and TRZ 10 mg/kg (2) DOX 4 mg/kg/2d was first injected intraperitoneally with a cumulative dose of 24 mg/kg and TRZ 1.66 mg/kg/2 d was injected with a cumulative dose of 10 mg/kg one week later	—	LVESV, LVEDD↑, LVEF, FS↓	Myocardial fibrosis, myocardial cell vacuolation, a large number of inflammatory cell infiltration	CK-MB, cTnI↑	[27, 30, 110, 111, 120]
Dox in combination with TRZ	Rat	(1) DOX 2.5 mg/kg/2d was first injected intraperitoneally with a cumulative dose of 15 mg/kg or 3.33 mg/kg/2 d with a cumulative dose of 20 mg/kg, and TRZ was injected with 3.33 mg/kg/2 d with a cumulative dose of 20 mg/kg 8 days later (2) DOX 2.5 mg/kg/2d was first injected intraperitoneally with a cumulative dose of 20 mg/kg, and TRZ was injected 3.3 mg/kg/2 d with a cumulative dose of 30 mg/kg 11 days later (3) DOX 3.3 mg/kg/2 d was first injected intraperitoneally with a cumulative dose of 20 mg/kg, and TRZ was injected 5 mg/kg/2d with a cumulative dose of 30 mg/kg 2 weeks later	—	LVESV, LVEDV↑, FS, LS↓	There is an extensive infiltration of macrophages around the cardiomyocytes	cTnI, NT-proBNP↑	[70, 105, 106, 112]
Radiation combined with targeted therapy							
Radiation in combination with TRZ	Rat	TRZ was given a single intraperitoneal injection of 6 mg/kg, followed by a single local irradiation of 15 Gy to the heart 2 hours later	—	—	Mitochondrial edema and vacuolar changes of myocardial cells	—	[76]
Radiation in combination with TRZ	Mice	A single local irradiation of 15 Gy or 20 Gy was given to the heart, followed 24 hours later by multiple intraperitoneal injections of TRZ 1.66 mg/kg/2 d with a cumulative dose of 10 mg/kg 24 hours later	Weight loss	LVPWT, IVST↑	Myocardial cells are disordered with vacuolated and adipose changes	—	[85, 99]

BNP: brain natriuretic peptide; CK-MB: creatine kinase isoenzyme; CRP: C-reactive protein; cTnI: cardiac troponin I; cTnT: cardiac troponin T; DOX: doxorubicin; d: day; EF: ejection fraction; ESV: end-systolic volume; FS: fractional shortening; HR: heart rate; ICIs: immune checkpoint inhibitors; IVST: interventricular septal thickness; IL-1*β*: interleukin-1*β*; IL-2: interleukin-2; IL-6: interleukin-6; IFN-*γ*: interferon-*γ*; LS: longitudinal strain; LDH: lactate dehydrogenase; LVDD: left ventricular end-diastolic dimension; LVEF: left ventricular ejection fraction; LVWT: left ventricular wall thickness; LVSP: left ventricular systolic pressure; LVSW: left ventricular-stroke work; LVESV: left ventricular end-systolic volume; LVEDV: left ventricular end-diastolic volume; LVIDD: left ventricular diastolic dimension; LVEDP: left ventricular end-diastolic pressure; LVESD: left ventricular end-systolic diameter; LVSWI: left ventricular-stroke work index; LVPDW: left ventricular end-diastolic posterior wall; LVPWT: left ventricular posterior wall thickness; NT-proBNP: N-terminal probrain natriuretic peptide; PD-1/CTLA-4: programmed cell death protein 1/cytotoxic T lymphocyte-associated antigen 4; RS: radial strain; TRZ: trastuzumab; TNF-*α*: tumor necrosis factor-*α*; w: week; 5-Fu: 5-fluorouracil; ±dp/dtmax: left ventricular pressure change rate; Ref: references.

## Data Availability

The data used to support the finding of this study are available from the corresponding author upon request.

## Abbreviations

BNP: Brain natriuretic peptide
CK-MB: Creatine kinase isoenzyme
CRP: C-reactive protein
CTnI: Cardiac troponin I
CTnT: Cardiac troponin T
DOX: Doxorubicin
EF: Ejection fraction
ESV: End-systolic volume
FS: Fractional shortening
FDA: Food and Drug Administration
HR: Heart rate
HER-2: Human epidermal growth factor receptor 2
ICIs: Immune checkpoint inhibitors
IVST: Interventricular septal thickness
IL-1*β*: Interleukin-1*β*
IL-2: Interleukin-2
IL-6: Interleukin-6
IFN-*γ*: Interferon-*γ*
LS: Longitudinal strain
LDH: Lactate dehydrogenase
LVDD: Left ventricular end-diastolic dimension
LVEF: Left ventricular ejection fraction
LVWT: Left ventricular wall thickness
LVSP: Left ventricular systolic pressure
LVSW: Left ventricular-stroke work
LVESV: Left ventricular end-systolic volume
LVEDV: Left ventricular end-diastolic volume
LVIDD: Left ventricular diastolic dimension
LVEDP: Left ventricular end-diastolic pressure
LVESD: Left ventricular end-systolic diameter
LVSWI: Left ventricular-stroke work index
LVPDW: Left ventricular end-diastolic posterior wall
LVPWT: Left ventricular posterior wall thickness
NT-proBNP: N-terminal probrain natriuretic peptide
PD-L1: Programmed cell death ligand protein-1
PD-1/CTLA-4: Programmed cell death protein 1/cytotoxic T lymphocyte-associated antigen 4
RS: Radial strain
RIHD: Radiation heart damage
TRZ: Trastuzumab
TNF-*α*: Tumor necrosis factor-*α*
5-FU: 5-Fluorouracil
±dp/dtmax: Left ventricular pressure change rate.
